# Active multiband varifocal metalenses based on orbital angular momentum division multiplexing

**DOI:** 10.1038/s41467-022-32044-2

**Published:** 2022-07-25

**Authors:** Ruixuan Zheng, Ruhao Pan, Guangzhou Geng, Qiang Jiang, Shuo Du, Lingling Huang, Changzhi Gu, Junjie Li

**Affiliations:** 1 Beijing National Laboratory for Condensed Matter Physics, Institute of Physics, Chinese Academy of Sciences, Beijing, 100190 China; 2 School of Physical Sciences, CAS Key Laboratory of Vacuum Physics, University of Chinese Academy of Sciences, Beijing, 100049 China; 3 School of Optics and Photonics, Beijing Engineering Research Center of Mixed Reality and Advanced Display, Beijing Institute of Technology, Beijing, 100081 China; 4 Songshan Lake Materials Laboratory, Dongguan, Guangdong, 523808 China

**Keywords:** Nanophotonics and plasmonics, Metamaterials

## Abstract

Metalenses as miniature flat lenses exhibit a substantial potential in replacing traditional optical component. Although the metalenses have been intensively explored, their functions are limited by poor active ability, narrow operating band and small depth of field (DOF). Here, we show a dielectric metalens consisting of TiO_2_ nanofins array with ultrahigh aspect ratio to realize active multiband varifocal function. Regulating the orbital angular momentum (OAM) by the phase assignment covering the 2π range, its focal lengths can be switched from 5 mm to 35 mm. This active optical multiplexing uses the physical properties of OAM channels to selectively address and decode the vortex beams. The multiband capability and large DOFs with conversion efficiency of 49% for this metalens are validated for both 532 nm and 633 nm, and the incidence wavelength can further change the focal lengths. This non-mechanical tunable metalens demonstrates the possibility of active varifocal metalenses.

## Abstract (web summary)

A dielectric metalens consisting of ultrahigh aspect ratio TiO_2_ nanofins array is demonstrated to realize active multiband varifocal functionality. By regulating the orbital angular momentum, the focal length can be switched from 5 mm to 35 mm with large DOFs.

## Introduction

Active multiband varifocal is one of the lenses’ important functions in optical applications. Traditionally, the imaging system requires complex mechanical deformation or displacement as well as heavy lenses to achieve zoom, but their large footprint prevents optical systems from becoming miniaturized. Metasurfaces composed of subwavelength antenna arrays promote the development of light optical devices^1^. The flourishing researches of metasurfaces have been extensively applicated in the field of waveplates^2,3^, holographic imaging^4–7^, optical information encryption^8,9^, and quantum communications^10–12^. In particular, metalenses, kinds of metasurfaces, have great application potential as a means to replace and surpass traditional optical lenses.

Unlike traditional optical lenses, which rely on the geometric curvature of the surface, metalenses are constructed of subwavelength artificial structures that provides refractive index gradients to regulate electromagnetic (EM) waves^13^. All properties of EM waves can be flexibly modulated by adjusting the configurations, such as wavelength^14,15^, polarization^16–19^, phase^20–22^, wave front^23–25^, and spin state^26^. And more than one wave property can also viable simultaneous control with a single cell capacitating multifunctional operations^27,28^. Metalenses have the advantages of rich functions^29,30^ and small size, which are expected to achieve the miniaturization of optical lenses and complex functions. This has attracted great attention of researchers, and they have devoted great enthusiasm and research works to realize various functions of metalenses. But, the realization of the great important varifocal function is still confronted with vicious difficulties, such as the poor ability of active regulation^31–33^, narrow working band^34^, and small depth of field (DOF)^35,36^. At present, they can actively control the optical field by refractive index control^5,37,38^, material transformation^31^, electromagnetic resonance control^39,40^, and electrodeformation^41^. However, it is still unable to achieve the active varifocal, especially, in the visible band, because of the single mode of regulation and limited processing capacity.

In recent years, optical information coding techniques based on orbital angular momentum (OAM) have been developed to achieve flexible active regulation. The OAM associates with the spatial phase profile of the vortex beam^42–44^, which can be described by *exp(ilφ)*, offering a promising route for achieving active metasurfaces^45–47^. Different from spin angular momentum (SAM) with only two values of ± *ħ* that can be encoded, an infinite number*l*for the OAM can be theoretically used as a flexible controllable degree of freedom^48^. This physical property greatly improves the dimension of active regulation in the additional phase of EM waves. Moreover, the modulation of light field based on Pancharatnam Berry (PB) phase is a simple and effective means, which has developed into a mature and effective coding methods. Combined with the PB phase modulation ability of symmetry-breaking antennas^47,49,50^, the metalenses can discretionarily realize selective modulation of vortex beams with OAMs. The orthogonal relation between each vortices ensures less crosstalk between the functions, greatly boosting the effective and reliable selection of the channels, which also promises an effective guarantee for the multiplexing^47,51^. Although the functions of optical information encryption and information transmission have been realized based on OAM^46^, few studies have been focused on the preparation of lenses with flexible varifocal capability.

In addition, in order to achieve the great multiband conversion capability covering the visible band, it is necessary to design and select the appropriate size for the material. At the same time, the multiband metalenses consisting of high refractive index dielectric nanofins, especially, high aspect ratio organizations, have substantial potential to replace the conventional hulking lens. Accurate and stable fabrication of the structures is essential to achieving active multiband metalenses, but the nanofabrication limitations also impede the commercial applications of the metalenses.

In this work, an active multiband varifocal dielectric metalens (VDM) based on OAMs are realized by disposing of titanium oxide (TiO_2_) nanofins. This optical multiplexing uses the physical properties of vortex beams that carry independent OAM channels to selectively address and decode the incident light. The TiO_2_ have high refractive index *n*, and so it is extremely suitable uses in visible light to introduce a proper forward scattering phase at defined positions along the interface. The selected size meets the requirements of 532 nm and 633 nm with both conversion efficiencies of about 50% by digital simulation of the finite-element difference time-domain (FDTD) method. Due to the subwavelength sizes being smaller than the diffraction limit of photolithography, electron beam lithography (EBL) is widely used to fabricate the nanofins. The TiO_2_ configurations are fabricated by atomic layer deposition (ALD) on a transparent quartz (SiO_2_) substrate, which ensures the nanofins with the dense and smooth texture. As-formed VDM have the 0.5 mm diameter consisting of more than 1.6 million nanofins with an ultrahigh aspect ratio of 10.7, demonstrating the ability and feasibility of large-area fabrication. Experimental demonstration of VDM verifies great focusing spots at pre-design locations and the doughnut-shaped light intensity distributions. Simultaneously, four focal lengths are switched from 5 mm to 35 mm by changing the OAMs carried by the incident vortex source. By adjusting the wavelength of the incidence, the VDM will modulate the electromagnetic wave to dedicate the focus at adjacent positions, which demonstrates the multiband working capability. Therefore, our approach in this work will provide real opportunities for active multiband varifocal of metalenses, which is expected to apply to the fields of 3D imaging, integrated optics and vortex optics.

## Results and discussion

### The design methodology and simulations of VDM

**Fig. 1:**
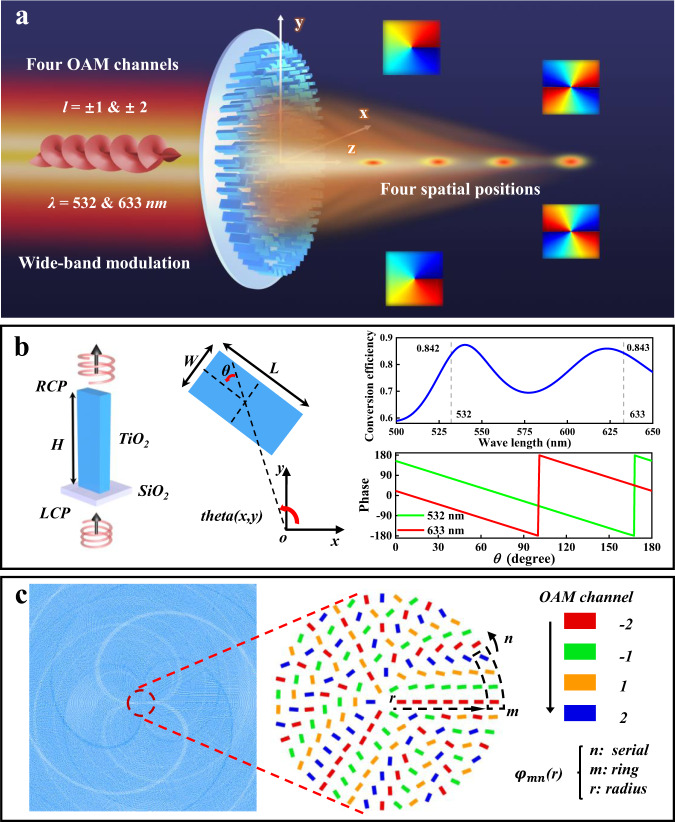
Function diagram and design principle of the VDM. **a** Schematic of the configuration and the functions with multiband varifocal of the VDM. **b** The left is the unit cell diagram and layout schematic, and the middle displays the relative positions between the center of the circle and rotation angle of the unit nanofin, where the coordinate axis means the center of the circle and the *θ* is the clockwise rotation angle of the unit nanofin. The right shows the rotation mode of nanofin covering 2π phase and the relationships between the rotation angle and the phase at 532 nm and 633 nm, respectively. **c** The map shows the details of arrangement of each nanofins of VDM to satisfy OAMs decoding.

The VDM consists of TiO_2_ nanofins with different rotation angles, which are used to modulate the PB phase of the incidence. As shown in Fig. 1a, different circles are allocated in certain methods to decode vortex beams with different OAMs. The four focuses can be observed from the transmitted side, and the focal position is on the *z* axis with the VDM center as the origin of the coordinate axis. In order to select suitable feature sizes of the nanofin, finite difference time domain (FDTD) simulations were applied to calculate the cross-polarized transmission of nanofins. Figure 1b exhibits the dimension diagram of unit nanofin with height *H* = 900 nm, length *L* = 178 nm, and wide *W* = 84 nm, which also indicates the arrangement of the x-y coordinate plane. According to Jones matrix, the cross-polarized components of the transmitted wave will carry PB phase, which equals to *φ* = *+2θ*, where ‘φ’ and ‘*+*’ represent the value of PB phase and the sign of the phase shift for LCP incidence, *θ* represents to the rotation angle. The nanofin can be modulated to cover 2π phase by its own rotation, as shown in the right illustration in Fig. 1b. It also shows that the relationships between the modulated phase and the rotation angle of the nanofin, where the phase changes are nearly twice of rotation angle for both 532 nm and 633 nm incidence. While the transmission conversion efficiency of the nanofin is also displayed, which can reach 84.2% at 532 nm and 84.3% 633 nm for left circular polarization (LCP) incidence in simulation. The configuration based on the rotation angle *θ* can realize the selection and control of OAMs. The VDM combined with the abilities of focal lens and spatial phase plate, which can decode the OAMs and focus to several points. Thus, the phase shifts of designed VDM can be calculated by following expression:

1 $$\varphi \left(r\right)\,=\,\frac{2{{\pi}}}{\lambda }\left(f-\sqrt{{r}^{2}+{f}^{2}}\right)+l\,{\cdot }\,{{{{{\rm{theta}}}}}}\left(x,y\right)$$

where *λ, f, and l* are the incident wavelength, focal length, and topological charge, respectively. The first term of Eq. 1 determines the focal function, while the second term is the spatial phase. Take the OAMs as the modulated channel, we set *l* *=* ± 2, ± 1 with focal lengths of ${f}_{i}$ (channel *i* = −2, −1, 1, 2) in sequence, where *i* represents OAM modes. Therefore, the phase distribution of the VDM with OAM multiplexing are shown as the follow functions:

2 $$\varphi \left(r\right)={\sum }_{{mn}}{\varphi }_{{mnl}}\left(r\right),\,r={mp},{np}=2\pi r$$

3 $${\varphi }_{{mnl}}(r)=\left\{\begin{array}{c}\frac{2\pi }{\lambda }({f}_{-2}-\sqrt{{r}^{2}+{{f}_{-2}}^{2}})-2\cdot {{\mbox{theta}}}(x,y),\,l=-\!2,\,{{{{{\mathrm{mod}}}}}}({{\mbox{n}}},\,4)=1\\ \frac{2\pi }{\lambda }({f}_{-1}-\sqrt{{r}^{2}+{{f}_{-1}}^{2}})-1\cdot {{\mbox{theta}}}(x,y),\,l=-\!1,\,{{{{{\mathrm{mod}}}}}}({{\mbox{n}}},\,4)=2\\ \frac{2\pi }{\lambda }({f}_{1}-\sqrt{{r}^{2}+{{f}_{1}}^{2}})+1\cdot {{\mbox{theta}}}(x,y),\,l=1,\,{{{{{\mathrm{mod}}}}}}({{\mbox{n}}},\,4)=3\hfill\\ \frac{2\pi }{\lambda }({f}_{2}-\sqrt{{r}^{2}+{{f}_{2}}^{2}})+2\cdot {{\mbox{theta}}}\left(x,y\right),\,l=2,\,{{{{{\mathrm{mod}}}}}}({{\mbox{n}}},\,4)=0\hfill\end{array}\right.$$

where n is the serial number of the nanofins in each circle, m is the serial number of the circle and the configuration step *p* = 0.3 μm. In order to show the designed by Eqs. 2 and 3 clearly, Figs. 1c and S1b shows the phase map and rotation layout of VDM, where each circle values of each rotation angle *θ* are marked. Fig. S1b shows the arrangement for nanofins, in which four colors mark each channel of their modulated OAMs. Varifocal metalens can be created by interleaving cells from different rotation rules, which means the whole same color local metasurface could achieve the specific decoding focus corresponding to the OAM. Based on this map, the multi-channel VDM can effectively focus the incidence on different spatial positions according to the topological charge *l*, realizing the ability to work as different numerical apertures (NA) lens.

**Fig. 2:**
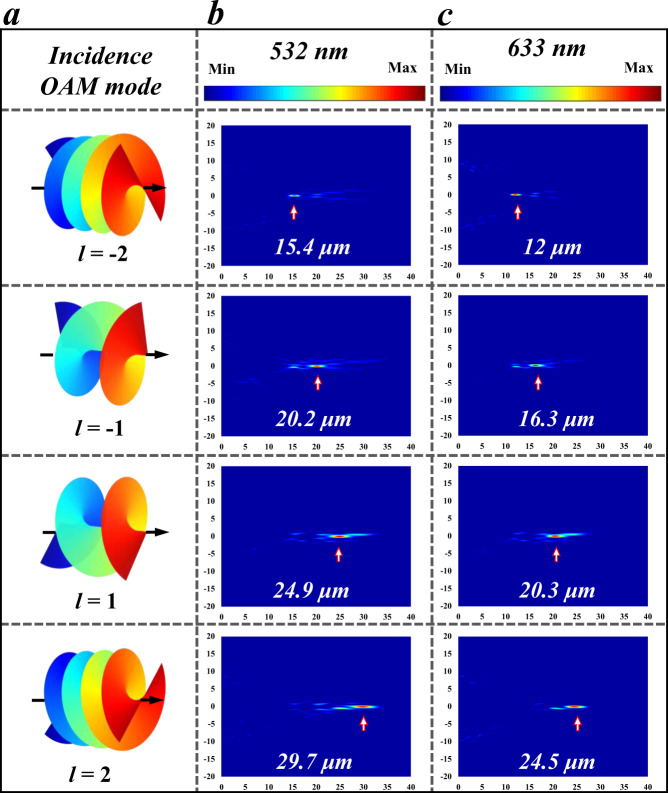
Simulation results for varifocal VDM of different incidence modes and wavelengths. **a** From up to down are the schematics of the vortex phase distribution with topological charge of −2, −1, 1, and 2, in sequence. The light field distributions of **b** 532 nm and **c** 633 nm incidences for different OAMs, where the white arrows mark the focal point positions.

For the sake of verifying the functions of the designed VDM, simulations of a VDM with a 50 μm diameter are carried out. To better satisfy the function of the spatial phase plate, the arrangement of VDM is optimized into a circular configuration. Where the interval of the structure within the circle is 300 nm that can obtain well OAM selection ability. As shown in Fig. 2, four focuses of the VDM implements the OAM dependent varifocal function. Vortex beams carrying different OAMs hold different spatial phase distributions, as shown in the schematic diagram of Fig. 2a. Owing to the conservation of OAM, only the configuration mode with an inverse topological charge (-*l*) can recover the fundamental spatial mode with a relatively stronger transmission distribution, which ensures that each focus could be decoded at 15 μm, 20 μm, 25 μm, or 30 μm by certain incident topological charge. It can be clearly seen that bright spots are generated in the designed spatial locations. In the case of 532 nm excitation, the focal lengths are 15.4 μm, 20.2 μm, 24.9 μm, and 29.7 μm as the nonzero OAM changes from −2 to 2, respectively, as shown in Fig. 2b. Importantly, a multiband response-ability can also be observed in Fig. 2c, where the focal lengths are ranged from are 12 μm, 16.3 μm, 20.3 μm to 24.5 μm, respectively. The output focuses are close to the designed lengths, which accord with the characteristics of a chromatic aberration lens. Therefore, the focuses can be adjusted in a range by changing the incident wavelengths. Moreover, the focusing efficiency of OAM is considered and the detector in simulation is slightly larger than the spot size to ensure adequate collection. The simulations reveal 12.76%, 12.23%, 12.22%, and 11.66% for the four positions, which means the total efficiency is about 48.87%. Meanwhile, the focusing effects for some wavelengths between 500 nm and 650 nm are shown in Fig. S2.

### VDM fabrication and characterization

**Fig. 3:**
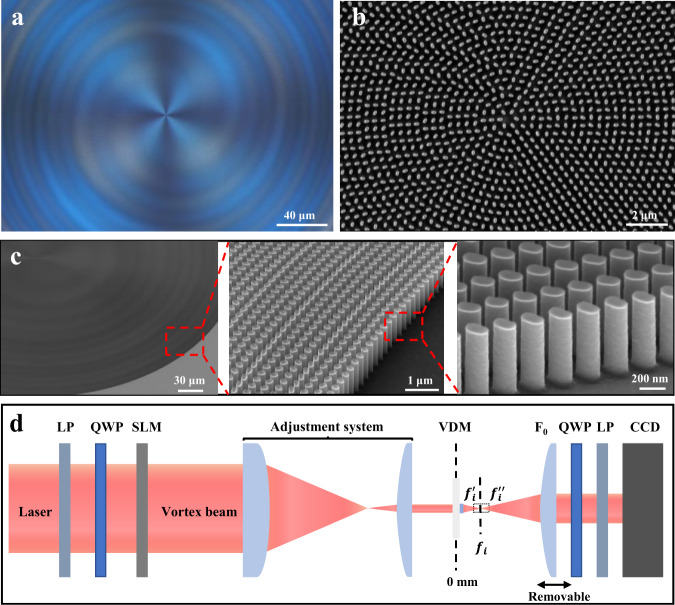
Fabricated devices and experimental setup. **a** Top view optical photo of the VDM center. **b** Detailed planform SEM image of the VDM center. **c** Configuration SEM images of the nanofins array at the margin of the VDM with increasing the resolution. **d** Optical setup for the varifocal focus reconstruction of OAM-dependent, LP, linear polarizer, QWP, quarter-wave plate, SLM, spatial light modulator, VDM, varifocal dielectric metasurface.

The simulations show the VDM with focal lengths at micrometer scale. However, the practical application needs that the VDM has a relatively large area and a longer focal length. Thus, we further fabricate a VDM with a diameter of 0.5 mm consisting of more than 2.6 million blocks. This VDM can achieve the better varifocal function that the focal lengths change from 5 mm to 35 mm. Figure 3a shows a top view optical photo of VDM center, and its structural color indicates that the nanofins are in a uniform shape over a large scale and visible vortex fringes can already be seen. The scanning electron microscopy (SEM) image of the center VDM, and the angular arrangement of each nanofin can be clearly seen (Fig. 3b). More fabricated VDM images are provided in Fig. S3, including an optical top view and a 52° SEM of the complete VDM, which show excellent large-size processing capability. EBL and ALD are used in fabricating that can keep great machining uniformity. The detailed processing process can be obtained in the section of experimental methods. Further, the high-definition SEM images of VDM edges with different magnifications are presented in Fig. 3c, in which that TiO_2_ nanofins not only maintain a high aspect ratio of 10.7 (*H*/*W*), but their surface morphology also has vertical walls and smooth texture. Even though the deviations of size and edges of nanofins can be observed from the manufacturer, their conversation efficiency can still remain at about 80%, as shown in Fig. S4. The optical setup for the reconstruction of OAM-dependent focuses is presented in Fig. 3d, in which a spatial light modulator (SLM) was used to dynamically generate vortex beams with different OAMs. The size of the incident beam is shrunk to the area of the VDM by an adjustment system. And a charge-coupled detector (CCD) has been introduced to capture the field distribution behind the VDM. To attest the varifocal capability, four vortex beams with helical mode index of OAM = −2, −1, 1, 2 are applied to illuminate on side of the VDM and four focuses can be observed from the other side.

**Fig. 4:**
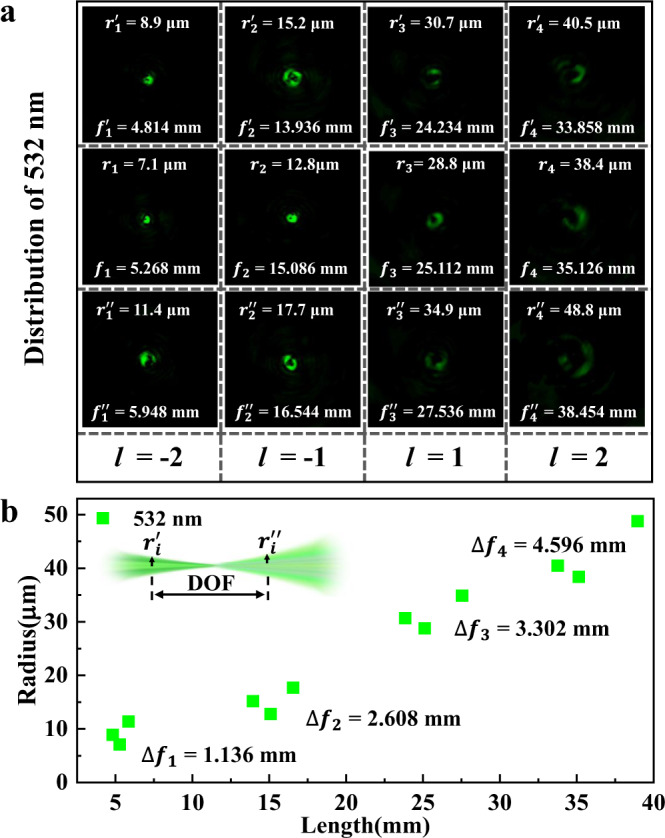
Experimental focal spots, radii, and the DOF of the VDM at 532 nm. **a** Collected field distribution, focal length, and radii of 532 nm for adjacent four focal planes with the OAM of −2, −1, 1, and 2. **b** Depict of the statistical results for the focal length, radii, and the DOF of the focal point for 532 nm.

The actual measurements of the prepared VDM at 532 nm laser incident with different OAMs are shown in Fig. 4. It can be observed that the focal spots for different OAMs always demonstrate a doughnut shape when the incident light with a wavelength of 532 nm (Fig. 4a), which is a typical field distribution of vortex beams with nonzero OAMs. Here, the focal spots (${f}_{i}$) are determined by the field distribution with the smallest bright point. And the DOF can be calculated by *f*_*i*_″ − *f*_*i*_′, which are the spatial distance of the focal point that can maintain a doughnut shape at the front (*f*_*i*_′) and back (*f*_*i*_″) locations of each focus. With the nonzero OAM changes from −2 to 2, the focal spots can be found at *f*_*-2*_ = 5.268 mm, *f*_*−1*_ = 15.086 mm, *f*_*1*_ = 25.112 mm and *f*_*2*_ = 35.126 mm, respectively. These experimental results show great consistency with the presupposed focal lengths. The radius (*r*) of the focal points is further gotten by the radius doughnut-like light intensity distributions, and the approximate range of the focuses are marked with white dash circles. The radii of the focal spots are *r*_*−2*_ = 7.1 μm, *r*_*−1*_ = 12.8 μm, *r*_*1*_ = 28.8 μm and *r*_*2*_ = 38.4 μm, respectively. The radii and DOF for the incidence to 532 nm wavelength are summarized in Fig. 4b. The DOF for the focuses are $\varDelta {f}_{-2}$ = 1.136 mm, $\varDelta {f}_{-1}$ = 2.608 mm, $\varDelta {f}_{1}$ = 3.302 mm and $\varDelta {f}_{2}$ = 4.596 mm, in sequence, indicating the fabricated VDM is a large DOF metalens. The intensity profile at the focal position shown in Fig. S5 also confirms that the light intensity distribution presents a good circular distribution.

The actual measurements of the prepared VDM at 532 nm laser incident with different OAMs are shown in Fig. 4. It can be observed that the focal spots for different OAMs always demonstrate a doughnut shape when the incident light with a wavelength of 532 nm (Fig. 4a), which is a typical field distribution of vortex beams with nonzero OAMs. Here, the focal spots (${f}_{i}$) are determined by the field distribution with the smallest bright point. And the DOF can be calculated by ${f}_{i}^{{\prime} {\prime}} -{f}_{i}^{\prime}$, which are the spatial distance of the focal point that can maintain a doughnut shape at the front (${f}_{i}^{{\prime} }$) and back (${f}_{i}^{{\prime} {\prime} }$) locations of each focus. With the nonzero OAM changes from −2 to 2, the focal spots can be found at *f*_*−2*_ = 5.268 mm, *f*_*−1*_ = 15.086 mm, *f*_*1*_ = 25.112 mm, and *f*_*2*_ = 35.126 mm, respectively. These experimental results show great consistency with the presupposed focal lengths. The radius (*r*) of the focal points is further gotten by the radius doughnut-like light intensity distributions, and the approximate range of the focuses are marked with white dash circles. The radii of the focal spots are *r*_−*2*_ = 7.1 μm, *r*_*−1*_ = 12.8 μm, *r*_*1*_ = 28.8 μm, and *r*_*2*_ = 38.4 μm, respectively. The radii and DOF for the incidence to 532 nm wavelength are summarized in Fig. 4b. The DOF for the focuses are $\varDelta {f}_{-2}$ = 1.136 mm, $\varDelta {f}_{-1}$ = 2.608 mm, $\varDelta {f}_{1}$ = 3.302 mm and $\varDelta {f}_{2}$ = 4.596 mm, in sequence, indicating the fabricated VDM is a large DOF metalens. The intensity profile at the focal position shown in Fig. S5 also confirms that the light intensity distribution presents a good circular distribution.

**Fig. 5:**
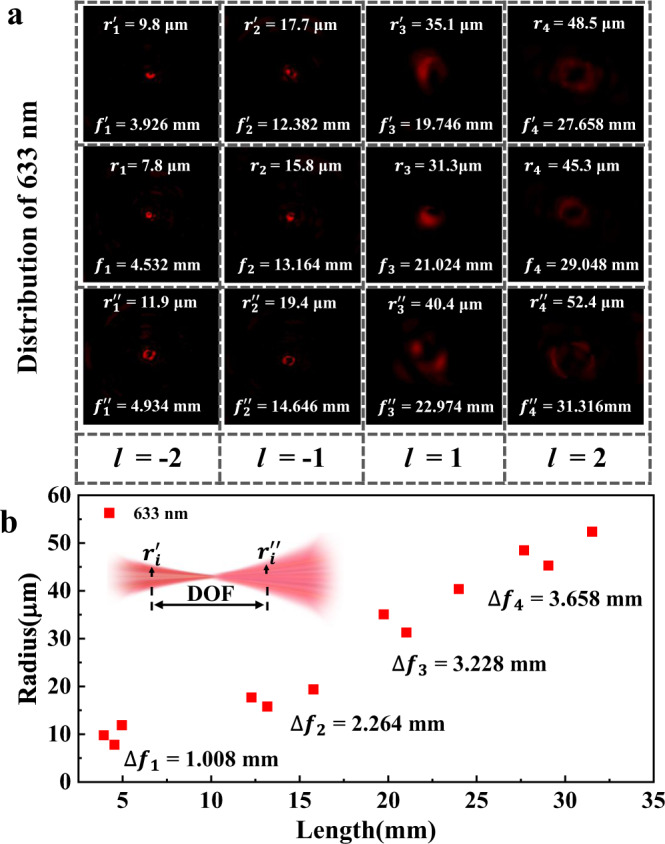
Experimental focal spots, radii, and the DOF of the VDM at 633 nm. **a** Collected field distributions, focal length, and radii of 633 nm for adjacent four focal planes with the OAM of −2, −1, 1, and 2. **b** Depict of the statistical results for the focal length, radii, and the DOF of the focal point for 633 nm.

To verify an ideal multiband optical modulation ability of as-fabricated VDM, the light distribution on the x-y cross section for the 633 nm incidence is demonstrated in Fig. 5a. Similar to the 532 nm incidence, four focuses with focal lengths of 4.532 mm, 13.164 mm, 21.024 mm and 29.048 mm can be observed, respectively. Each focal point has a radius of r_1_ = 7.8 μm, r_2_ = 15.8 μm, r_3_ = 31.3 μm and r_4_ = 45.3 μm. As shown in Fig. 5b, except for the changes of the focal lengths, the DOF of the focuses are $\varDelta {f}_{1}$ = 1.008 mm, $\varDelta {f}_{2}$ = 2.264 mm, $\varDelta {f}_{3}$ = 3.228 mm, and $\varDelta {f}_{4}$ = 3.658 mm. There are certain differences between the lengths obtained from 532 nm and 633 nm incidences. Notably, a similar trend can also be found in the 633 nm incident light. With the increase of propagation distance, the decrease of NA leads to the weakness of focus intensity and the increase of DOF. It is reasonable that the increase of the radius and the reduction in focal brightness origin from the dissipation in the transmission of light.

The field distribution at the plane of defocus position is also considered. In Fig. S6, the light intensity at the focal positions (*f*_*1*_ and *f*_*2*_) and the non-focal position (*f*_*0*_) are compared, and it can be clearly seen that the middle figure with *f*_*0*_ = 10 mm is no significant brightness. Where the light of Fig. S6b is due to the error caused by the scattering of the environment and CCD integration. This proves that the light field energy can be localized near the focal points of our design according to the phase arrangement. Some noises in the figures are due to the imperfect modulation efficiency of the elements in the large distance optical system or the little misalignment of the light. Moreover, the dynamic changes of the light field distributions near the focal points are recorded in Movie 1 and Movie 2 (Supplementary information) under the 532 nm incident carrying −2 and −1 OAMs. The doughnut-shaped distribution of light can be observed in the videos. As the focal lengths change from small to large, the spot sizes go through a process from relatively large to a minimum and then to large. Simultaneously, the doughnut-shaped light fields pass from appearing to brightest to vanishing, which convincingly demonstrates that the VDM is a large DOF metalens. In spite of some deviations in the measurement, the experimental zooming function is as expected, which fully verifies the feasibility of multiband VDM for multi-channel OAMs. To summarize, we have demonstrated a design method of a multiband VDM composed of TiO_2_ nanofins for multiplexing varifocal in momentum space. The VDM is designed on the regulation of PB phase, which have combined the ability of focal lens and spatial phase plate. Thus, the OAM can be used as a new controllable degree, which enables flat metalenses the ability of multi-channel light selective focus. In addition, the broad operating band VDM achieves good focusing and zooming capabilities in both 532 nm and 633 nm lasers. This gives us confidence that if we further optimize the structures and system, better performance of active varifocal metalenses will be achieved in the future. And we believe that our multiband OAM-multichannel VDM contributes to a substantial advance towards non-mechanical switching imaging and 3D imaging devices.

## Methods

### Simulations of TiO_2_ metasurface

Relying on the crosstalk-free selectivity of multiple OAMs, we have designed a multi-channel multiband VDM. To quantify the phase retardation of light transmitted through TiO_2_ nanofins, electromagnetic simulations of subwavelength nanofins arranged in a square lattice were performed using the FDTD. The effective refractive index is obtained from the fundamental modes while the power coupling into higher-order waveguide modes is ignored. We specify the optical dispersion parameters of TiO_2_ nanofins from ellipsometry measurements realized on epitaxially grown TiO_2_ thin-film on a single-side polished silicon wafer, which used in the simulation. To determine the size of the nanofin, perfectly matched layer (PML) conditions in the direction of the light propagation and periodic boundary conditions along all the in-plane directions were used. Then, the use of PML in-plane boundary conditions mimic a subwavelength array of desired nanostructures. As a result, the conversion efficiency and the phase modulation relationship caused by different in-plane rotation angles of TiO_2_ fins were numerically characterized, as shown in the Fig. 2b, c.

### Fabrication of nanostructures

In this work, quartz cleaned in acetone, isopropanol (IPA), and deionized water in sequency is used to hold the 3D nanofins. Secondly, the polymethyl methacrylate (PMMA) resist is spun on a clean quartz plate to obtain a thick enough photoresist film and baked at 180 °C for 1 min. Successively, the patterns are exposed using electron beam lithography system (6300FS, JEOL), and then the patterns are obtained by developing in a mixture of MIBK and IPA (1:3). Next, the ALD process of TiO_2_ is carried out in a home-built system. H_2_O is the source of O, and tetrakis (dimethylamino) titanium (TDMAT) precursor is used as the Ti source without chlorine contamination and achieved the required vapor pressure by heating the chamber. Meanwhile, the continuous flow of Ar carried gas throughout the development. After ALD process, a dry etching process is executed in the ICP-RIE system (Plasmalab System 100 ICP180, Oxford) with a mixed reactive gas of CHF_3_ to remove the TiO_2_ film on the top of the resist. Finally, another dry etching process with Oxygen plasma is applied to remove the residual resist. A scanning electron microscope (SEM) (Helios 600i, FEI) is used to characterize the morphology of the samples.

### Optical setup for the characterization of varifocal dielectric metasurface

The primary optical components used for characterizing the OAM-selective and multiplexing varifocal metasurface are shown in Fig. 3d. The laser beams at wavelengths of 633 nm and 532 nm (Thorlabs) propagates through a multiband linear polarizer. Then, the LCP beams were produced by phasing linearly polarized light through a quarter of a paddle. To generate the vortex beams needed to decode the different channels, the LCP beams were dynamically modulated by a spatial light modulator (SLM, Daheng Optics) to imprint a helical wavefront onto the optical beam via the phase function of a spiral phase plate. Next, the incident OAM beams were relayed to the multi-channel sample surface through an adjustment system, where the adjustment system is used to conjugate the SLM plane to the VDM plane. And the system consists of a lens (*f* = 25 cm) and an objective lens (20×/0.4) to compress the facula size to match the sample. Thereafter, the incident OAM beams were focused by the VDM with four focal lengths. The reconstructed light distributions from the VDM were collected by a transmissive objective lens (L_0_, 10×/0.25) which was mounted on a three-dimensional translation stage to determine the spatial position of different focal points, and imaged on a pixelated charge-coupled device camera (CCD, Nanobox).

## Supplementary information

Supplementary information

Description to Additional Supplementary Information

Movie 1

Movie 2

## Data Availability

All data supporting the findings of this study are available within the article and its Supplementary Information files and from the corresponding authors upon reasonable request.

## Code availability

All code will be provided upon request to the authors. Please address your requests for data and code to the corresponding authors.
