# Supplementary material for: Active multiband varifocal metalenses based on orbital angular momentum division multiplexing
Source: Nat Commun. 2022 Jul 25;13:4292. doi: 10.1038/s41467-022-32044-2 (PMC9314414; doi:10.1038/s41467-022-32044-2)
Supplement: Supplementary file 1 — Supplementary information [file 41467_2022_32044_MOESM1_ESM.pdf]

Additional information

Supplementary information

Active Multiband Varifocal Metalenses Based on Orbital Angular Momentum Division Multiplexing

Ruixuan Zheng [1,2], Ruhao Pan[1], Guangzhou Geng [1], Qiang Jiang [4], Shuo Du [1], Lingling Huang [4], Changzhi Gu [1,2] ✉, Junjie Li [1,2,3] ✉

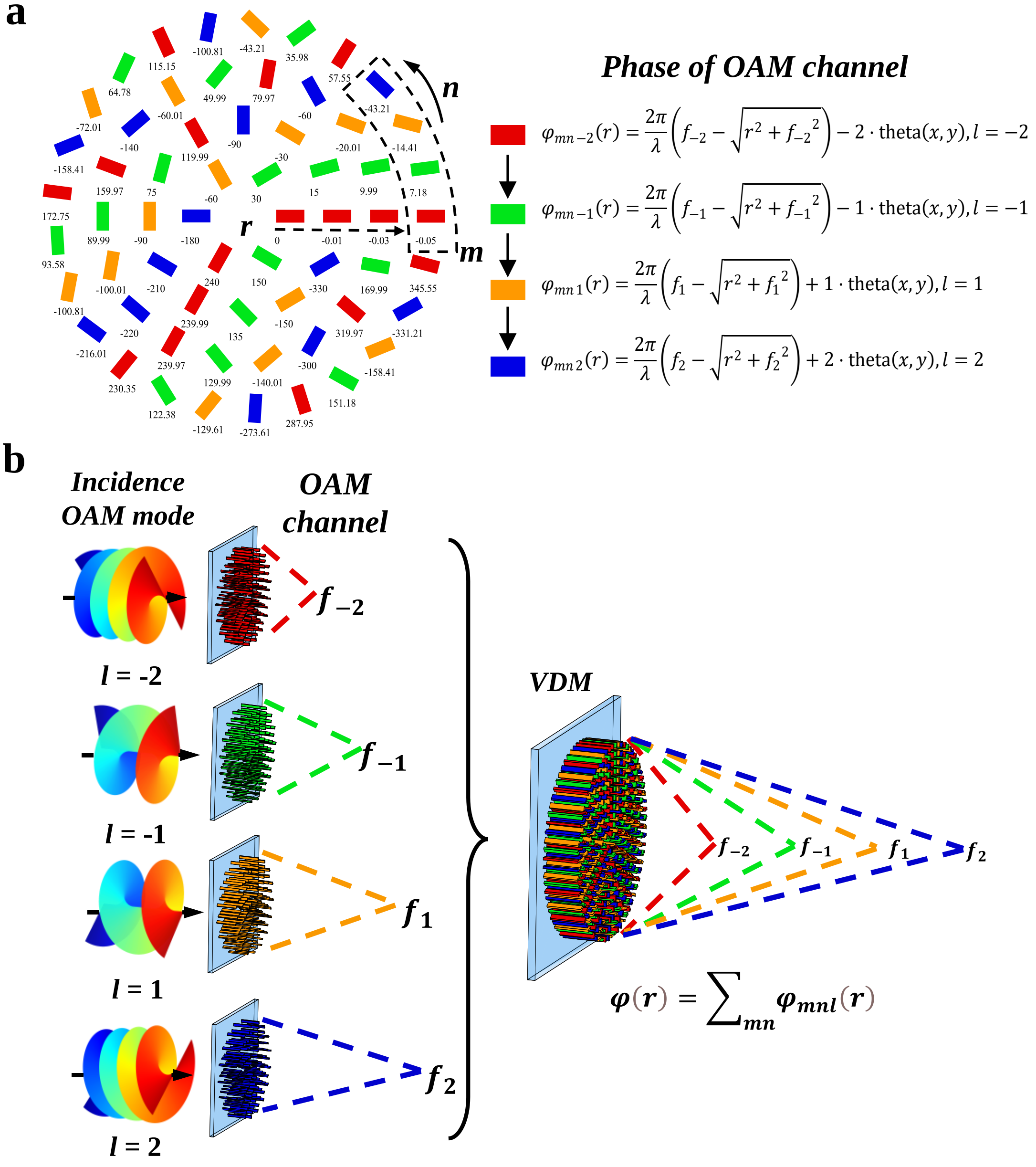

Fig. S1 Design principle of varifocal by space division multiplexing. (a) The rotation angle θ values of the center nanofins to meet the phase requirements for four channels. (b) Multichannel design principle of VDM.

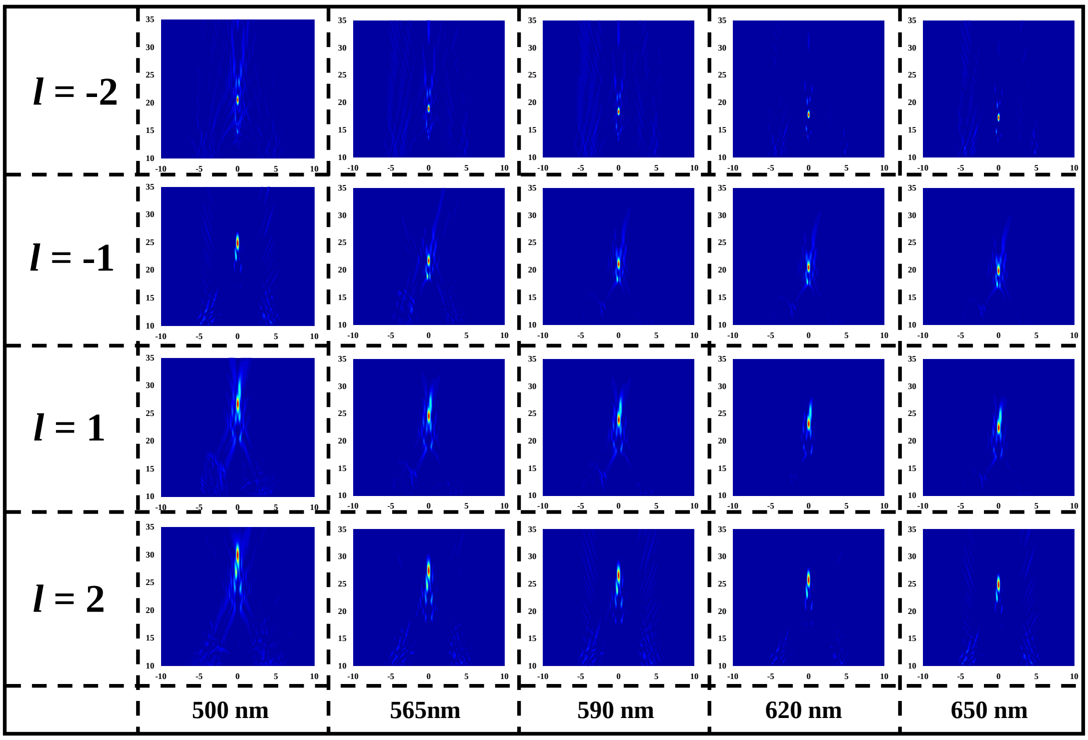

Fig. S2 The focusing effect at different incident wavelengths between 500 – 650 nm.

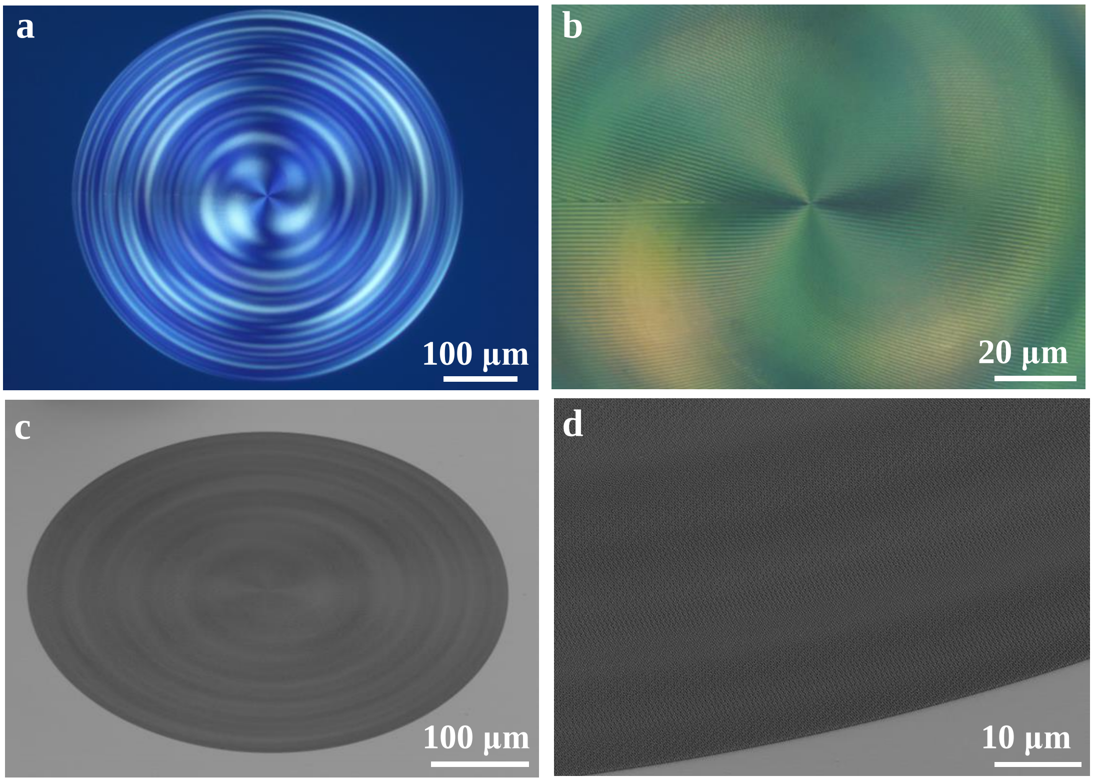

Fig. S3 Optical and SEM images with different magnifications of the VDM.

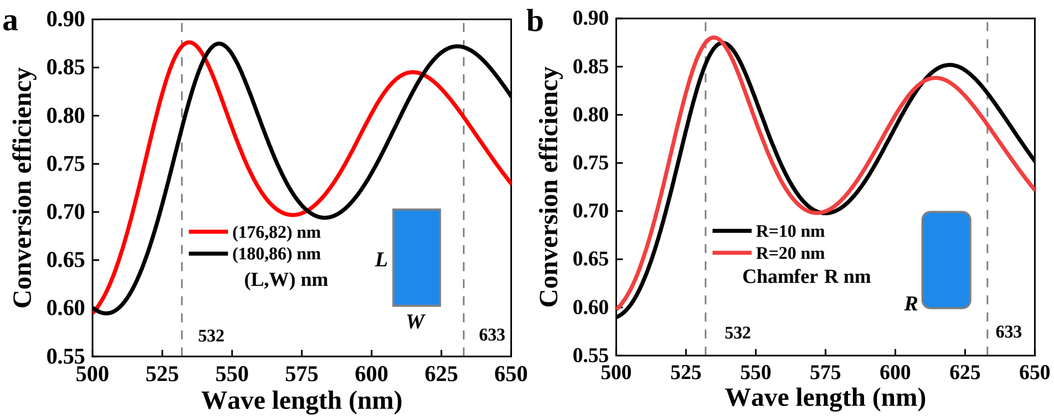

Fig. S4 Effect of size deviation of nanofin on transmission conversion efficiency. The curves of the structure with (a) larger or smaller size and (b) the chamfered corners (R). As for inset of (a), the length (L) and width (W) of the top of the nanofin are marked; the illustration in (b) is marked as the chamfering of radius (R) for the apex angle of the nanofin.

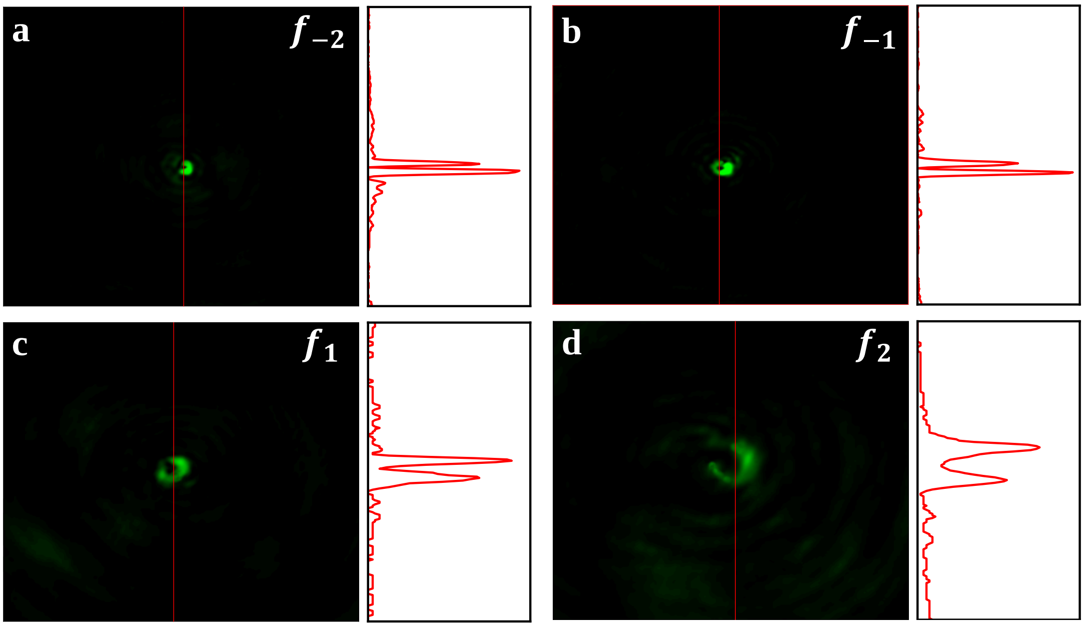

Fig. S5 Light intensity distribution of four focuses for 532 nm. (a), (b), (c) and (d) show the light field distribution at the focusing position for OAM = -2, -1, 1 and 2, respectively. The curves on the right are the normalized light intensity distribution of the section.

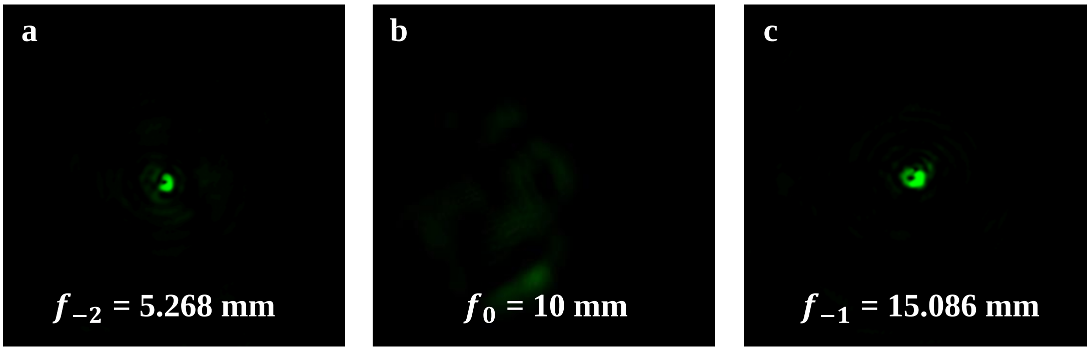

Fig. S6 Light intensity distribution. (a) and (c) show the light field distribution at the focusing position for OAM = -2 and -1, respectively, and (b) shows the light field distribution at the non-focusing position at 10 mm.
