# Supplementary material for: Active multiband varifocal metalenses based on orbital angular momentum division multiplexing
Source: Nat Commun. 2022 Jul 25;13:4292. doi: 10.1038/s41467-022-32044-2 (PMC9314414; doi:10.1038/s41467-022-32044-2)
Supplement: Supplementary file 2 — Description to Additional Supplementary Information [file 41467_2022_32044_MOESM2_ESM.pdf]

**Description of Additional Supplementary Files**

**Movie 1**. Dynamic distributions of the light field near the focal point for 532 nm carrying -2 OAM. The video shows that the intensity distributions of the light field change with the focal length f from small to large.

**Movie 2**. Dynamic distributions of the light field near the focal point for 532 nm carrying -1 OAM. The video shows that the intensity distributions of the light field change with the focal length f from small to large.
